# Prevalence of and risk factors for curable sexually transmitted infections on Bubaque Island, Guinea Bissau

**DOI:** 10.1136/sextrans-2019-054351

**Published:** 2020-04-28

**Authors:** Giovanna Cowley, Gregory Milne, Eunice Teixeira da Silva, Jose Nakutum, Amabelia Rodrigues, Hristina Vasileva, David Mabey, Bart Versteeg, Anna Last

**Affiliations:** 1 London School of Hygiene and Tropical Medicine, London, UK; 2 Pathobiology and Population Sciences, Royal Veterinary College, Hatfield, Hertfordshire, UK; 3 Simao Mendes Hospital, Bissau, Guinea-Bissau; 4 Hospital Regional de Bubaque Marcelino Banca, Bubaque, Guinea-Bissau; 5 National Institute of Public Health, Guinea Bissau, Bissau, Guinea-Bissau; 6 Clinical Research, London School of Hygiene and Tropical Medicine, London, UK

**Keywords:** epidemiology (general), risk factors, genital tract infection, Africa

## Abstract

**Objectives:**

Complications from sexually transmitted infections (STIs) can result in severe morbidity and mortality. To date, no STI population studies have been conducted on the Bijagos Islands, Guinea Bissau. Our objective was to estimate the prevalence of and identify risk factors for *Chlamydia trachomatis (Ct), Neisseria gonorrhoea (Ng), Mycoplasma genitalium (Mg), Trichomonas vaginalis (Tv)* and *Treponema pallidum (Tp*) on Bubaque, the most populated island.

**Methods:**

A cross-sectional survey was conducted on the island of Bubaque among people aged 16–49 years. Participants were asked to answer a questionnaire on STI risk factors, to provide urine samples (men and women) and vaginal swabs (women) for PCR testing for *Ct, Ng, Mg and Tv,* and to provide dry blood spots for *Tp* particle agglutination assays. Data were analysed to estimate the prevalence of STIs and logistic regression was used to identify risk factors.

**Results:**

In total, 14.9% of participants were found to have a curable STI, with the highest prevalence being observed for *Tv* (5.9%) followed by *Ct* (3.8%), *Ng* (3.8%), *Mg* (1.9%) and *Tp* (0.8%). Significant risk factors for having any STI included being female, younger age and concurrent partnership. Having had a previous STI that was optimally treated was a protective factor.

**Conclusions:**

This study demonstrates that there is a considerable burden of STI on the Bijagos Islands, stressing the need for diagnostic testing to facilitate early detection and treatment of these pathogens to stop ongoing transmission. Moreover, these results indicate the need to conduct further research into the STI burden on the Bijagos Islands to help inform and develop a national STI control strategy.

## Introduction

An estimated 357 million cases of curable sexually transmitted infections (STIs) occur each year worldwide.^1^ *Chlamydia trachomatis (Ct*), *Neisseria gonorrhoea (Ng), Trichomonas vaginalis (Tv*) and *Treponema pallidum (Tp*) account for a large proportion of these^1^ and, more recently, *Mycoplasma genitalium (Mg*) has been recognised as a significant STI.^2^ Although often initially asymptomatic, complications from these curable STI can result in severe morbidity and mortality.^3–5^

STIs disproportionately affect low-income countries and prevalence estimates are usually higher for women.^6^ As laboratory‐based testing is frequently unavailable in low-income countries, STIs are usually treated through syndromic management.^6^ However, most STIs remain asymptomatic constituting a large reservoir for ongoing infection. Moreover, when left undiagnosed and untreated these can result in complications including pelvic inflammatory disease that can lead to infertility, ectopic pregnancy, miscarriage and stillbirth in women and epididymo-orchitis and subsequent subfertility in men.^4^ The inability to detect asymptomatic infections using laboratory-based diagnostics also limits the availability of epidemiological data that can help to better understand the burden of STI. Studies are therefore needed to understand STI transmission so that targeted intervention and control programmes can be developed to reduce the burden of infection.^6^

Guinea Bissau is one of the least-developed countries in the world.^7^ Data on the prevalence of curable STI and their risk factors are scarce. STI research in Guinea Bissau has largely focused on selected groups, including women with urogenital symptoms,^8–10^ women giving birth at the National Referral Hospital,^11^ the police^12^ and military professionals,^13^ providing little information on STI in the general population.

The Bijagos Islands lie off the coast of Guinea Bissau; no data on STI are available for this region. The population is highly mobile, has matriarchal elements to its structure and people continue to follow traditional religious practices.^14 15^ These unusual cultural aspects mean that sexual behaviour, patterns of STI transmission and health-seeking behaviour, may not be typical. Furthermore, the Bijagos Islands have been a site for mass drug administration (MDA) with azithromycin for trachoma control.^16^ Azithromycin is an antibiotic that is effective against a number of STI^17^; however, the impact of MDA on STI prevalence is not known in this setting.

Here, we conducted a cross-sectional study of STI prevalence on Bubaque, the most populated of the Bijagos islands of Guinea Bissau. We aimed to estimate the prevalence of *Ct*, *Ng, Mg*, *Tv* and *Tp* and to identify risk factors associated with these infections.

## Methods

### Ethical approvals

This study was conducted in accordance with the declaration of Helsinki. Ethical approvals were given by the Comitê Nacional de Ética e Saúde, Guinea Bissau (ref 022) and the ethics committee of the London School of Hygiene and Tropical Medicine (LSHTM) (ref 11701). Written consent (signature or a thumb print) was provided by all participants.

### Study design

A cross-sectional study of people aged between 16 and 49 living on Bubaque Island, Guinea Bissau was conducted over a 12week period in 2016.

A target sample size of 500 participants was calculated to give sufficient power based on an estimated prevalence of urogenital *Ctt* of 10% in adults, a 95% CI of 1.96 (α=5%), a degree of absolute precision of 3% (d=0.03) and a design effect for cluster surveys taken from previous urogenital chlamydia studies of 1.26.^18^ It was estimated that 150 households were needed to recruit 500 participants.

A probability-proportional-to-size sampling method was used, with the number of households randomly selected from each of the 23 villages weighted by population size. Eligibility criteria included age between 16 and 49 years, capacity to consent and ability to provide samples.

### Data collection

Trained fieldworkers used a structured questionnaire to conduct private interviews on socioeconomic factors (religion, education, income and marital status), sexual history (age of sexual debut, number of partners in 1 year, concurrent partners, previous STI and their treatment, contraception use, condom use) and symptoms (genital ulcer, urethral discharge or dysuria in men, and genital ulcer, vaginal discharge or lower abdominal pain in women). Participants reporting STI symptoms were referred to the local hospital for syndromic management. Participants provided dry blood spots (DBS) and urine samples and women also provided two self-taken high vaginal swabs (HVS) taken under nurse-guidance. Dry samples were stored on Bubaque at 4°C for a maximum period of 1 month until transfer to Bissau (−20°C) and then to the LSHTM, London (−80°C).

### Laboratory procedures

The BioChain DNA isolation kit (catalogue no. K5017100) was used to extract DNA from urine samples (men and women) and HVS (women) following the manufacturer's recommendations. Primer sets were adapted from the AmpliSens multiplex real-time PCR, which simultaneously detected *Ct*, *Ng*, *Mg* and *Tv*.^19^ PCR primer concentrations were optimised for the Rotor-Gene^TM^ 3000 platform and diagnostic validity was assessed using a panel of known-positive and negative samples of the 4 STI (Qnostics STI Evaluation Panel 01). The assay was then applied to all extracted DNA samples. A single positive sample defined an individual as having that STI.

DBS were tested using the *Tp* particle agglutination assay (TPPA, Fujirebio, Tokyo, Japan), according to the manufacturer’s protocol. Positive and negative controls were included in each plate. The results were interpreted by two technicians independently. Samples with discordant and/or indeterminate results were repeated. Repeated samples that gave indeterminate results were excluded from further analysis.

### Data analyses

STATA (V.15.1, StataCorp) was used to calculate STI prevalence, to perform statistical tests and multivariable regressions to determine risk factors for STI.

First, a logistic regression model of STI infection status and village was performed to determine if multivariable regression would need to be adjusted for the cluster sample methodology. Univariable logistic regression models of STI status and each potential risk factor were performed (adjusted for age and sex). Risk factors with p<0.10 in the univariable analysis were entered into a multivariable model. Risk factors with p<0.05 in the final model were deemed to be independent risk factors for STI.

## Results

### Participant characteristics

**Figure 1:**
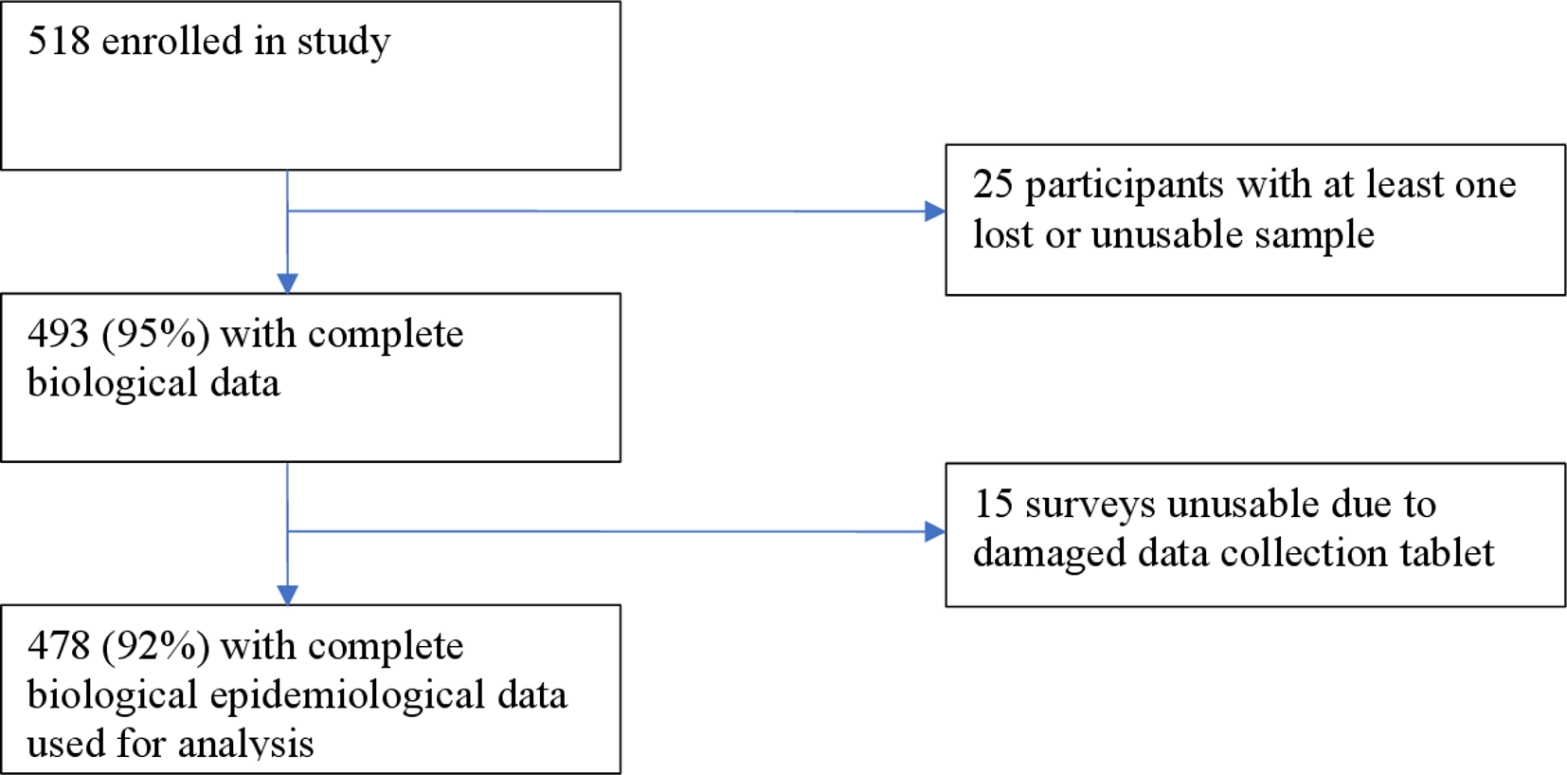
Flowchart indicating the number of participants included in data analysis from the study population.

Between May and July 2016, 518 participants from 150 households were enrolled into the study. Complete biological sample and epidemiological data (figure 1) were available for 478/518 (92%) participants, which was used to asses risk factors associated with having an STI. The median age of participants was 24 years (range: 16–59) and 59.2% of participants were women. The majority of participants (60.5%) had been to secondary school, 62.7% earned an income and 87.5% were married. On average, female participants had had 2.6 pregnancies and 21.6% of women were pregnant at the time of the study. Sexual debut had occurred under the age of 16 for 32.8% of participants. Of the 7.9% of participants who reported never having had sex, five were found to have an STI. A significant proportion of participants (29.9%) had never used condoms and while the majority of participants (62.3%) reported having had one sexual partner in the last year, 25.1% had more than one current partner. Characteristics are further described in online supplementary material, table S1.

10.1136/sextrans-2019-054351.supp1 Supplementary data

### STI prevalence

A complete dataset was available for 478 participants. The prevalence of STI was 21.9% for women, which was significantly higher than 4.6% for men (table 1) (OR=5.81, 95% CI 2.82 to 12.00, p<0.001). Even when HVS results were excluded and only prevalence of positive male and female urine samples were compared, women were still significantly more likely to have an STI (11.1% compared with 4.6%, OR 2.58, 95% CI 1.20 to 5.56, p=0.015). No male samples were positive for *Tv* and, after excluding *Tv* from estimates, STI were still more common in women (OR=3.01, 95% CI 1.42 to 6.39, p=0.004).

**Table 1:** STI prevalence and prevalence by sex

	Overall	Male	Female
**Any STI prevalence (%) among participants (n/N, 95% CI)**			
Any STI	14.9 (71/478)	4.6 (9/195)	21.9 (62/283)
	11.79 to 18.36	2.10 to 8.58	17.23 to 27.19
**STI prevalence (%) by type among participants (n/N, 95% CI)**			
STI type			
*Ct*	3.8 (18/478)	1.0 (2/195)	5.7(16/283)
	2.22 - 5.89	0.12 - 3.66	3.27 - 9.01
*Ng*	3.8 (18/478)	3.6 (7/195)	3.9 (11/283)
	2.25 to 5.89	1.47 to 7.26	1.96 to 6.85
*Mg*	1.9 (9/478)	0.5 (1/195)	2.8 (8/283)
	0.86 to 3.54	0.01 to 2.82	1.22 to 5.49
*Tv*	5.9 (28/478)	0 (0/195)	9.9 (28/283)
	3.93 to 8.36		6.68 to 13.98
*Tp*	0.8 (4/478)	0 (0/195)	1.4 (4/283)
	0.23 to 2.12		0.39 to 3.58

Ct, *Chlamydia trachomatis*; Mg, *Mycoplasma genitalium*; Ng, *Neisseria gonorrhoea*; Tp, *Treponema pallidum*; Tv, *Trichomonas vaginalis*.

Overall prevalence of STI by type was 5.9% for *Tv*, 3.8% for *Ct* and *Ng*, 1.9% for *Mg* and 0.8% for *Tp*. It is important to note that, unlike the molecular tests for other STI that detect current infection, the serological TPPA test for *Tp* cannot distinguish between current and previous infection or between syphilis and endemic treponematoses. It is therefore possible that the identified cases of *Tp* do not represent current infection with syphilis.

All STIs were more common in women. The prevalence of co-infection was 1.3% (6/478, 95% CI 0.46% to 2.71%): 2 *Ct/Mg*, 1 *Ct/Ng*, 1 *Ct/Tv*, 1 *Ng/Tv* and 1 *Ct/Tp*. Five (83.3%) of these co-infections occurred in women. Only 3.2% (2/62) of women with an STI reported symptoms, which was no different to the 3.3% (7/214) of women without an STI who reported symptoms. Men with an STI were significantly more likely to report symptoms than women (22.2% compared with 3.2% (*χ^2^* (1, n=71)=5.33, p=0.021).

### Risk factors for STI

A logistic regression model of STI infection status and village was non-significant (OR=0.97, 95% CI 0.92 to 1.03, p=0.33, n=488); therefore, multivariable regression did not require adjustment for the cluster sampling methodology.

Univariate analysis showed that age, sex and having concurrent partners were risk factors for STI. Having more than one partner in the last year also tended towards being a significant risk factor. Reporting optimal treatment of a previous STI (treated at a medical centre and partner treated) tended towards being a protective factor. Level of education, income, religion, marital status, age of sexual debut, previous STI, condom use, pregnancy and reporting of current symptoms of an STI were not significant risk factors.

Multivariable analysis showed that younger age, female sex and concurrent partnership were significant risk factors for STI. Having previously had an STI which was treated optimally (treated at a medical centre and partner treated) was a protective factor against being STI positive (table 2).

**Table 2:** Independent risk factors for STI in univariate and multivariate models

Risk factor	Category	N/N	STI positive (%)	OR (95% CI) univariate analysis*	P value	OR (95% CI) multivariate analysis	P value
Age (years)	16–19	27/140	19.3	2.14 (1.00 to 4.57)	0.022	2.88 (1.27 to 6.51)	
	20–25	19/120	15.8	1.44 (0.65 to 3.19)		1.89 (0.82 to 4.37)	0.005
	26–35	13/112	11.6	0.99 (0.42 to 2.31)		1.21 (0.50 to 2.92)	
	36+	12/106	11.3	1.0		1.0	
Sex	Female	62/283	21.9	5.80 (2.81 to 11.98)	<0.001	14.06 (6.0 to 35.69)	<0.001
Previous STI optimally treated	Yes	2/38	5.3	0.27 (0.07 to 1.27)	0.082	0.20 (0.04 to 0.91)	0.038
Concurrent sexual partner	Yes	18/120	15.0	4.56 (2.09 to 9.96)	<0.001	7.69 (2.09 to 24.2)	<0.001
Number of partners in 1 year	0	7/55	12.7	1.0	0.05	1.0	0.168
	1	49/298	16.4	0.81		0.77 (0.30 to 1.99)	
	>1	15/108	13.9	2.65		0.38 (0.06 to 2.34)	
Education	None	12/58	20.7	1.0	0.684		
	Primary	14/119	11.8	0.55 (0.23 to 1.24)			
	Secondary	45/289	15.6	0.82 (0.36 to 1.87)			
	Tertiary	0/12	0	–			
Income	Yes	5/80	6	0.84 (0.30 to 2.35)	0.744		
Religion	None	30/166	18.1	1	0.337		
	Catholic	23/145	15.9	0.85 (0.45 to 1.59)			
	Protestant	17/98	17.4	1.27 (0.62 to 2.58)			
	Muslim	1/43	2.3	0.15 (0.02 to 1.14)			
Married or living with partner	Yes	63/355	17.8	0.84 (0.35 to 2.01)	0.703		
Age of sexual debut<16	Yes	28/157	17.8	0.85 (0.55 to 1.32)	0.481		
Previous STI	Yes	11/91	12.1	1.10 (0.53 to 2.27)	0.807		
Condom use	Never	19/143	13.3	1	0.462		
	Sometimes	35/174	20.1	1.49 (0.78 to 2.85)			
	Usually	4/29	13.8	1.33 (0.40 to 4.47)			
	Often	13/75	17.3	1.40 (0.61 to 3.23)			
Number of pregnancies	0	18/66	27.3	1	0.537		
	1	23/97	23.7	0.92 (0.42 to 2.02)			
	2 to 4	12/60	20.0	0.85 (0.28 to 2.61)			
	>4	9/60	15.0	0.65 (0.17 to 2.44)			
Pregnant at time of study	Yes	9/61	14.8	0.56 (0.26 to 1.22)	0.143		
Current STI symptoms	Yes	4/19	21.1	1.85 (0.55 to 6.21)	0.317		

*For all other variables, the regression was adjusted for age and sex.

STI, sexually transmitted infection.

## Discussion

Here, we report the first data on curable STI collected in the general population in Guinea Bissau. Our findings indicate the need for an STI control programme.

We found 14.9% of study participants to have one or multiple curable STI. One year prior to our study, azithromycin, an antibiotic that has activity against *Ct, Ng, Mg* and *Tp*, had been distributed to the population as part of an MDA programme for trachoma elimination.^16^ In the Solomon Islands, a similar MDA campaign resulted in a 40% reduction in age-adjusted urogenital *Ct* prevalence.^20^ It is possible that STIs in our population have been similarly affected but unfortunately no data on STI prevalence prior to MDA is available for Bubaque. Longitudinal studies to assess changes in STI prevalence after MDA as well as azithromycin resistance patterns would be useful in characterising the impact of MDA in this context.

As would be expected, STI prevalence was mostly lower in our study than in previous studies from Guinea Bissau which estimated prevalence among individuals presenting to sexual health clinics. Prevalence of *Ct, Mg* and *Tv* among women in Bissau in our study was 5.7%, 2.8% and 9.9% compared with 12.6%, 7.7% and 20.4% in a previous study on symptomatic women.^10^ In contrast, *Ng* prevalence was higher in our study (3.9% vs 1.3%) but this is likely explained by more sensitive diagnostics (PCR compared with bacterial culture) used in our work. Indeed, another study of people presenting to an STI clinic in Bissau found an *Ng* prevalence of 17% in women and 38% in men,^8^ which is consistent with higher rates expected in those seeking medical assistance for symptoms. In an older study of women giving birth or aborting at the Simão Mendez Hospital in Bissau,^11^ *Mg* prevalence was higher than among women in our study (6.2% vs 2.8%). There may be a number of characteristics associated with attending the national referral hospital to give birth that explain this difference in results.

Although *Tv* was the most prevalent STI found in women, no cases were identified in male participants. Previous work from the Netherlands found *Tv* to be rare in men and not associated with symptoms.^21^ In contrast, a study from Mwanza showed it to be a common STI in symptomatic men.^22^ These contrasting findings are perhaps unsurprising given the different study populations. However, as infections are cleared quickly in men, *Tv* prevalence is likely to be low in unselected population studies such as ours.^23^

*Tp* was detected in only 0.8% of participants. Unlike molecular tests used to detect current infection for the other STI, the TPPA test used to diagnose *Tp* infection does not distinguish between current and previous infection or between syphilis and endemic treponematoses. It is therefore possible that these cases of *Tp* were acquired in the past. This is consistent with the fact that, whereas other STI mostly occurred in younger age groups, all positive *Tp* results occurred in women over 30. Previous work in police officers in Bissau suggests a downward trend in *Tp* prevalence from 4.5% in 1990 to 0.4% in 2010.^12^ Older women might have a residual positive TPPA test having acquired syphilis prior to a decline in prevalence or from exposure to yaws in childhood.

In our study, even after exclusion of *Tv,* being female was a significant risk factor for STI. This is a widely recognised phenomenon^1 24 25^ with a number of explanations. In other contexts, gender power imbalances prevent women from negotiating safe sex^24^ but in a matriarchal society, where women can make decisions about relationships,^14 15^ this is less likely to be a factor. On the other hand, having the power to change partner might increase the number of partners a woman has and therefore the risk of STI. A further reason is that women are more susceptible to pathogen entry due to greater trauma over a larger mucosal area during sex.^26^ Finally, higher rates of asymptomatic infections are likely to contribute to higher prevalence of STI in women. In this study, women with an STI were significantly less likely to report symptoms than men. Indeed, even after prompting with a description of potential symptoms, 97% of women found to have an STI denied having symptoms. The standard syndromic management approach used in this context would not detect these cases. This highlights the urgent need for cheap and practical screening methods to detect asymptomatic STI as untreated infections can result in severe sequelae and contribute to ongoing transmission.^4^

Younger age was also a risk factor for STI. Young people often engage in greater risk-taking behaviour and, in women, a less mature cervix allows for easier pathogen entry.^27^ Concurrent partnership is another risk factor that has previously been well described.^28^ Reporting having had a previous STI that was medically treated had no relationship with current risk of STI. However, if partners had also been treated, the risk of current STI was reduced. This supports the importance of partner notification strategies in breaking the cycle of STI transmission.^29^ Surprisingly, condom use was not associated with lower risk of STI although inconsistent or incorrect use may explain this result.^30^

Potential limitations of this study should be noted. First, Bubaque may not be representative of the region as a whole. Furthermore, sampling was done by household and prevalence may therefore be overestimated. If one member of a household had an STI, their included partner would also be more likely to have an STI. 32.8% of participants reported sexual debut under the age of 16 but under-16s were not eligible for inclusion, meaning data from this potentially important group is lacking. Although participant numbers were sufficient to determine prevalence and identify certain risk factors for STI, the study was likely underpowered to detect all relevant factors. Finally, questionnaire data are limited by recall bias and some answers may not have been truthful. Indeed 5 of 39 people who reported never having had sex tested positive for an STI.

In conclusion, this is the first study to document the prevalence of curable STI in a general population in this region. An estimated prevalence of 14.9% shows that STI are an important problem on Bubaque. We find that younger age, female sex and having multiple concurrent partners are significant risk factors for STI and our data demonstrate the importance of partner notification in reducing the risk of STI. We show that, in this context, most people with an STI do not report symptoms even after prompting. In the absence of screening programmes for asymptomatic infections, their STI would not be detected or treated, placing them at risk of developing complications and continuing the spread of infection. This indicates the need for accurate diagnostic methods that can help to detect and treat asymptomatic infections through the development of a national STI control programme. Overall, our results demonstrate a need for a national STI control programme in Guinea Bissau and pave the way for further epidemiological work to develop this.

**Key messages**

- Sexually transmitted infections (STI) are a significant problem on Bubaque Island and an STI control strategy is needed.
- Women on Bubaque Island are disproportionately affected by STI.
- Most people with an STI do not report symptoms, indicating the need for feasible screening methods to detect and treat asymptomatic infections.
