# Supplementary material for: Prevalence of and risk factors for curable sexually transmitted infections on Bubaque Island, Guinea Bissau
Source: Sex Transm Infect. 2020 Apr 28;97(1):51–5. doi: 10.1136/sextrans-2019-054351 (PMC7841470; doi:10.1136/sextrans-2019-054351)
Supplement: Supplementary data [file sextrans-2019-054351supp001.pdf]

Table S1 Baseline characteristics of 478 participants with complete data

**Characteristic**		n	%
Female		283	59.2
Age (years)			
	16-19	140	29.2
	20-25	120	25.1
	26-35	112	23.4
	36+	106	22.2
Education			
	None	58	12.1
	Primary	119	24.9
	Secondary	289	60.5
	Tertiary	12	2.5
Religion			
	No religion	190	39.8
	Catholic	145	30.3
	Protestant	98	20.5
	Muslim	44	9.2
	Declined to answer	1	0.2
Income			
	No income	178	37.3

	Wage	80	16.7
	Trader	220	46.0
Alcohol	Yes	182	38.1
Smoker	Yes	9	1.9
Married/ Living with partner		418	87.4
Previous pregnancy			
	0	60	21.2
	1	66	23.3
	2 to 4	97	34.3
	>4	60	21.2
Pregnant at time of study		61	21.6
Ever used contraception		295	61.7
Age of sexual debut			
	Reported to never have had sex	38	7.9
	<16	157	32.8
	16+	270	56.5
	Declined to answer	13	2.7
Condom use			
	N/A	38	7.9
	Never	143	29.9

	Sometimes	174	36.4
	Mostly	33	6.9
	Always	88	18.4
	Declined to answer	2	0.4
Concurrent partner*		120	25.1
Number of partners last year			
	0	55	11.5
	1	298	62.3
	>1	123	25.7
	Declined to answer	2	0.4
Previous STI**		91	19.0
Previous STI treated at medical centre		83	91.2
Previous STI optimally treated***		38	41.8
Current Symptoms****		19	4.0

*The participant reported having more than one current sexual partner

**The participant reported having been diagnosed with an STI in the past

*** The participant reported having had a previous STI, having had it treated and their partner having been treated

**** Participants reported at least one of the following symptoms: genital ulcer, urethral discharge or dysuria (men only), vaginal discharge or lower abdominal pain (women only)
